# Biophysical and functional properties of purified glucose-6-phosphatase catalytic subunit 1

**DOI:** 10.1016/j.jbc.2021.101520

**Published:** 2021-12-21

**Authors:** Derek P. Claxton, Emily M. Overway, James K. Oeser, Richard M. O'Brien, Hassane S. Mchaourab

**Affiliations:** Department of Molecular Physiology and Biophysics, Vanderbilt University, Nashville, Tennessee, USA

**Keywords:** glucose-6-phosphatase, endoplasmic reticulum, glucose homeostasis, diabetes, glycogen storage disease, membrane protein, β-C12M, n-dodecyl-β-D-maltopyranoside, CHS, cholesteryl hemisuccinate, EGFP, enhanced green fluorescent protein, ER, endoplasmic reticulum, F6P, fructose-6-phosphate, FSEC, fluorescence detection size-exclusion chromatography, G6P, glucose-6-phosphate, GSD, glycogen storage disease, hG6PC1, human glucose-6-phosphatase catalytic subunit 1, IMAC, immobilized metal affinity chromatography, LMNG, lauryl maltose neopentyl glycol, M6P, mannose-6-phosphate, mG6PC1, mouse glucose-6-phosphatase catalytic subunit 1, MOI, multiplicity of infection, P_i_, inorganic phosphate, POPC, 1-palmitoyl-2-oleoyl-glycero-3-phosphocholine, SEC, size-exclusion chromatography

## Abstract

Glucose-6-phosphatase catalytic subunit 1 (G6PC1) plays a critical role in hepatic glucose production during fasting by mediating the terminal step of the gluconeogenesis and glycogenolysis pathways. In concert with accessory transport proteins, this membrane-integrated enzyme catalyzes glucose production from glucose-6-phosphate (G6P) to support blood glucose homeostasis. Consistent with its metabolic function, dysregulation of *G6PC1* gene expression contributes to diabetes, and mutations that impair phosphohydrolase activity form the clinical basis of glycogen storage disease type 1a. Despite its relevance to health and disease, a comprehensive view of G6PC1 structure and mechanism has been limited by the absence of expression and purification strategies that isolate the enzyme in a functional form. In this report, we apply a suite of biophysical and biochemical tools to fingerprint the *in vitro* attributes of catalytically active G6PC1 solubilized in lauryl maltose neopentyl glycol (LMNG) detergent micelles. When purified from Sf9 insect cell membranes, the glycosylated mouse ortholog (mG6PC1) recapitulated functional properties observed previously in intact hepatic microsomes and displayed the highest specific activity reported to date. Additionally, our results establish a direct correlation between the catalytic and structural stability of mG6PC1, which is underscored by the enhanced thermostability conferred by phosphatidylcholine and the cholesterol analog cholesteryl hemisuccinate. In contrast, the N96A variant, which blocks *N*-linked glycosylation, reduced thermostability. The methodologies described here overcome long-standing obstacles in the field and lay the necessary groundwork for a detailed analysis of the mechanistic structural biology of G6PC1 and its role in complex metabolic disorders.

Operating at the metabolic hub of gluconeogenesis and glycogenolysis, glucose-6-phosphatase maintains interprandial blood glucose homeostasis by catalyzing hydrolysis of glucose-6-phosphate (G6P) to glucose and inorganic phosphate (P_i_) (1, 2, 3). A resident multicomponent complex of the endoplasmic reticulum (ER) membrane, glucose-6-phosphatase coordinates the function of a G6P/P_i_ exchanger (SLC37A4) to facilitate transport of substrate from the cytosol into the ER lumen (4, 5, 6) and its hydrolysis by the catalytic subunit (G6PC) (7). Although three isoforms (G6PC1–3) have been identified with unique tissue distribution (2), G6PC1 is expressed predominantly in the liver and kidney where it functions as the gatekeeper of glucose production (2, 8, 9, 10). An extensive body of literature suggests that numerous metabolites and hormones regulate *G6PC1* gene expression (11), including insulin (repression) and glucagon (stimulation) (2, 12). Moreover, *G6PC1* mRNA and glucose-6-phosphatase activity are elevated in animal models of both type 1 and type 2 diabetes (1, 3, 11). Consequently, an increase of G6PC1 activity has been implicated in the pathology of diabetes by driving hepatic glucose production, a major contributor to hyperglycemia (13, 14, 15, 16, 17, 18). On the other hand, aberrant mutations in G6PC1 that reduce or abrogate enzyme activity cause glycogen storage disease (GSD) type 1a, which is characterized primarily by severe hypoglycemia (19, 20).

While its role in metabolic disease makes G6PC1 an attractive therapeutic target, the conspicuous absence of efficient heterologous expression and purification methodologies precludes a detailed molecular understanding of its structure and function. Until now, G6PC1 models have been predicted from low-resolution biochemical experiments and activity measurements within the context of intact microsomes. Cloning, sequence analysis, and proteolysis studies predict a 357-amino acid glycoprotein with nine transmembrane helices (21, 22). Large loops that project into the ER lumen support formation of the active site containing a consensus phosphatase sequence motif including strictly conserved Arg and His sidechains (23, 24). The groundbreaking computational structure prediction algorithm AlphaFold 2 has generated a 3D template for G6PC1 possessing a fold consistent with the sequence analysis and biochemical studies (25, 26), affording a preliminary model for which to interpret a wealth of experimental and clinical data. Nevertheless, the model, which captures a single predicted conformation, requires experimental validation.

The stable isolation of catalytically active G6PC1 in sufficient quantities is a prerequisite to these structural studies. Historically, limited natural abundance (0.1% of total liver protein) and sensitivity to solubilizing detergents have prevented successful purification of G6PC1 (3, 27, 28, 29, 30). Informed by a series of complementary screens that outline the expression/activity relationship of detergent solubilized enzyme, we describe for the first time the biophysical and functional characterization of the mouse G6PC1 ortholog purified from Sf9 insect cell membranes into lauryl maltose neopentyl glycol micelles. The isolated enzyme, glycosylated at Asn96, demonstrates robust catalysis of G6P and bears biochemical properties consistent with those previously observed in intact microsomes. Furthermore, kinetic modeling combined with thermostability analysis establishes a direct correlation between structural and catalytic stability, uncovering a fundamental role for lipids in G6PC1 structure and function.

## Results

### General approach and screening methodology

Previous attempts to purify G6PC1 have been marred by intractable yields and catalytic instability of the enzyme following solubilization of hepatic microsomes with harsh detergents (for example, sodium cholate and various Triton surfactants) (27, 29, 30, 31). To overcome this hurdle, we employed a multifaceted methodological approach to identify appropriate conditions for detergent extraction of G6PC1 overexpressed in a heterologous host with the goal of maintaining phosphohydrolase activity. In this screening approach, we correlated G6PC1 expression levels ascertained by fluorescence detection size-exclusion chromatography (FSEC) (32) with measurements of P_i_ production from G6P hydrolysis mediated by detergent-solubilized HEK293S cells following transfection of adherent culture.

**Figure 1:**
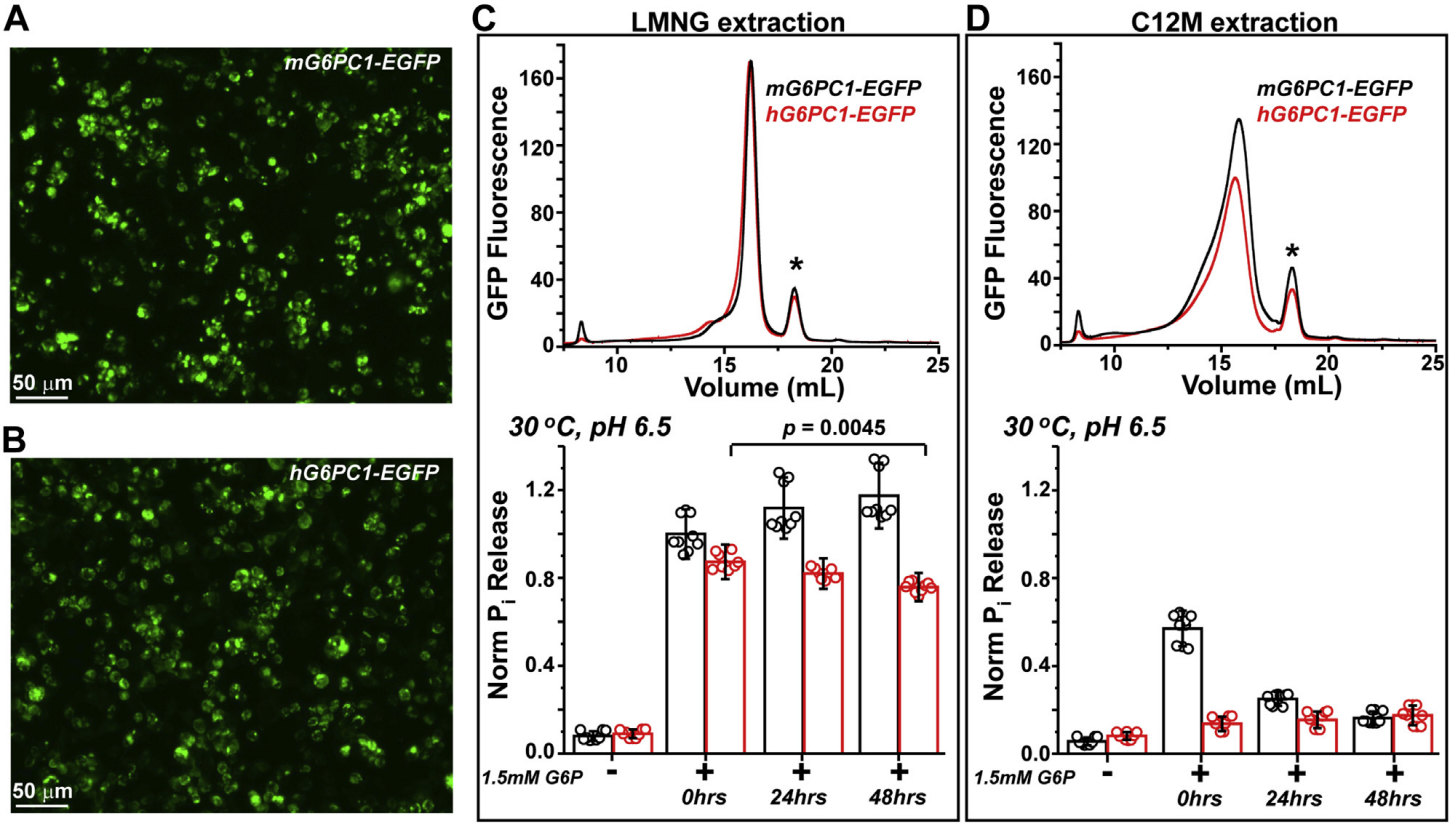
**Detergent screening of G6PC1 orthologs expressed in mammalian cells.***A* and *B*, false color images of cell epifluorescence following transfection of the mouse (*A*) and human (*B*) G6PC1-EGFP fusions. (*C*, *top panel*) FSEC analysis of LMNG-solubilized G6PC1 displays favorable homogeneous traces relative to those obtained from β-C12M extraction (*D*, *top panel*). The profiles shown are representative traces. The peak highlighted by the *asterisk* arises from minor cleavage of the fusion protein during solubilization. G6PC1 solubilized in LMNG retains functional activity (*C*, *bottom panel*). An unpaired *t* test of relative nmol P_i_ released from hG6PC1-EGFP solubilized in LMNG at t = 0 h (0.87 ± 0.08) and t = 48 h (0.76 ± 0.06) was used to compare the means and determine the *p* value shown. In contrast, G6P hydrolysis of G6PC1 solubilized in β-C12M decays rapidly (*D*, *bottom panel*). The free P_i_ concentration of untransfected controls in the presence of substrate was nearly identical to the free P_i_ carried over from the G6P stock (1.8 nmol, <1% of total P_i_ content), data not shown. EGFP, enhanced green fluorescent protein; FSEC, fluorescence detection size-exclusion chromatography; LMNG, lauryl maltose neopentyl glycol.

The WT mouse and human orthologs (mG6PC1 and hG6PC1, respectively), which share 89% sequence identity, were expressed as fusion proteins encoding a C-terminal EGFP tag to confirm expression and facilitate the FSEC analysis. Importantly, FSEC profiles also report the propensity of target proteins to either degrade or aggregate upon detergent solubilization. In general, nonuniform, multi-Gaussian elution peaks are the hallmarks of destabilized proteins, whereas the more favorable homogeneous chromatograms are suggestive of monodispersed and properly folded proteins (32). Preliminary FSEC screening that employed representatives of distinct detergent classes identified lauryl maltose neopentyl glycol (LMNG) and n-dodecyl-β-D-maltopyranoside (β-C12M) as lead candidates for extraction of G6PC1 (Fig. S1). In subsequent experiments, we compared the biophysical and functional properties of mG6PC1 and hG6PC1 when solubilized in these two popular, nonionic detergents (Fig. 1).

### G6PC1 solubilized in LMNG micelles supports structural and functional integrity

Epifluorescence images of HEK293S cells acquired 48 h post transfection indicated robust expression of the fusion constructs (Fig. 1, *A* and *B*). Following harvest, the cells were treated with either 5 mM (0.5% w/v) LMNG or 40 mM (2% w/v) β-C12M and the solubilized material injected onto a Superose6 Increase 10/300 G/L column for FSEC analysis (Fig. 1, *C* and *D*, top panels). The mG6PC1-EGFP and hG6PC1-EGFP constructs displayed similar elution profiles with respect to the solubilizing detergent (compare red and black traces within a panel). However, extraction with β-C12M produced broad, heterogeneous traces relative to the monodisperse profiles of LMNG-solubilized enzyme (compare top panels in Fig. 1, *C* and *D*). This result suggested the formation of aggregated species in β-C12M proteomicelles.

In order to determine the effect of the detergents on phosphohydrolase activity, we measured P_i_ release upon incubation of the detergent solubilized enzymes with substrate under biochemical conditions previously shown to promote catalysis in microsomes (3, 9, 33, 34). To account for differences in G6PC1 expression level across multiple transfection experiments (n = 3), the relative activity of the solubilized enzymes was determined by normalizing the P_i_ released to the integral of the FSEC elution peak (*i.e.*, the area under the curve), a surrogate reporter of enzyme concentration. In correlation with the homogeneous FSEC profiles, LMNG solubilization supported G6P hydrolysis as observed by an approximately tenfold increase in free P_i_ (Fig. 1*C*, lower panel). Remarkably, mG6PC1-EGFP activity remained stable even after 48 h post extraction. A notable, yet subtle decline in hG6PC1-EGFP activity in LMNG proteomicelles was associated with the rise of aggregated population(s) over the time course of the experiment (Fig. S2). In contrast, the initial (t = 0) phosphohydrolase activity of mG6PC1-EGFP was attenuated ∼40% in β-C12M proteomicelles relative to LMNG and nearly nonexistent at the 48 h time point relative to the 0 mM G6P control (Fig. 1*D*, lower panel). Furthermore, the activity of hG6PC1-EGFP was only slightly above background at all time points when solubilized in β-C12M. Collectively, the FSEC and activity assays indicated that solubilization with LMNG was superior to β-C12M in stabilizing folded G6PC1 and maintaining catalytic function.

### LMNG extraction of functional mouse G6PC1 from Sf9 insect cell membranes

Informed by the HEK293S transfection studies, we explored the feasibility of G6PC1 expression and extraction from Sf9 insect cells following baculovirus transduction. While hG6PC1 has been expressed previously in Sf9 cells, attempts to solubilize the enzyme with a variety of detergents reportedly failed (35). We focused on screening mG6PC1 since the FSEC and activity assays (Figs. 1 and S2) indicated that hG6PC1 is less stable.

**Figure 2:**
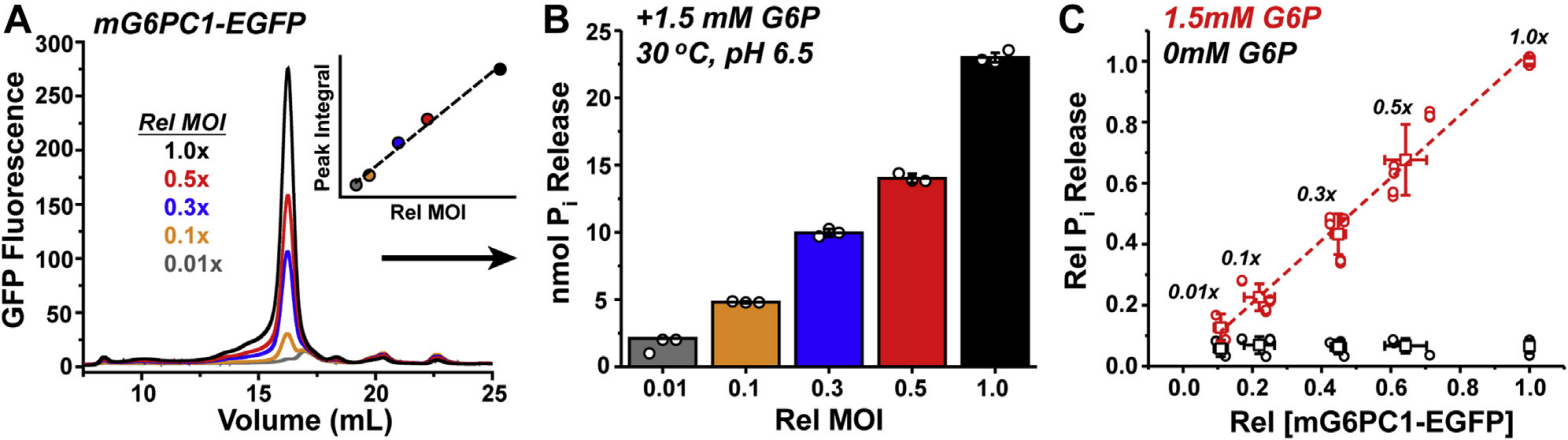
**Expression and activity of mG6PC1-EGFP from Sf9 cells transduced with baculovirus.***A*, FSEC traces report a linear increase in mG6PC1-EGFP expression with viral load (Rel MOI) and show a strong correspondence with activity measurements (*B*). The *inset* in (*A*) plots the FSEC peak area as a function of Rel MOI (slope = 0.92) and corresponds with Sf9 epifluorescence quantification shown in Fig. S3. The data in (*A* and *B*) are shown as a representative dataset. Activity measurements in (*B*) show the total nmol P_i_ released after subtraction of the 0 mM G6P control, which contained 2.00 ± 0.12 nmol P_i_ in the background. The analysis was performed in triplicate and is shown with error bars to describe the standard deviation from the mean. *C*, phosphohydrolase activity linearly correlates with mG6PC1-EGFP expression level (slope = 1.03). The standard deviation from the mean is shown as error bars from three independent measurements performed in triplicate. The data in (*B* and *C*) were obtained from mG6PC1-EGFP extracted from Sf9 membranes with LMNG. *Dashed lines* are linear regressions of the data. FSEC, fluorescence detection size-exclusion chromatography; G6P, glucose-6-phosphate; MOI, multiplicity of infection.

The WT mG6PC1-EGFP construct was introduced systematically into Sf9 suspension cells by titrating recombinant baculovirus with the goal of establishing the relationship between enzyme expression level and phosphohydrolase activity. Fusion protein expression levels were controlled by transducing Sf9 cells with five distinct concentrations of titered baculovirus, referred to as the relative multiplicity of infection (Rel MOI, see Experimental procedures for more details). The FSEC and G6P hydrolysis assays were performed on LMNG-solubilized enzyme 24 h post infection. Under these conditions, the baculovirus titration resulted in expression levels of mG6PC1-EGFP that were linear with respect to the viral load (Rel MOI) according to cell epifluorescence (Fig. S3) and FSEC analysis (Fig. 2*A*). Moreover, the FSEC elution profiles indicated that solubilized mG6PC1-EGFP was homogeneous (Fig. 2*A*), mirroring the results from the HEK293S expression studies. In accordance with the expression pattern, mG6PC1-EGFP solubilized with LMNG from Sf9 membranes demonstrated catalytic activity that tracked with baculovirus dosage (Fig. 2*B*). Indeed, P_i_ release in the presence of substrate was linearly correlated with the mG6PC1-EGFP expression level derived from the FSEC analysis (Fig. 2*C*). This result suggested that mG6PC1-EGFP heterologously expressed in Sf9 cells could be solubilized by LMNG while retaining phosphohydrolase activity.

### Catalytic properties of purified mouse G6PC1

Encouraged by these results, we sought to purify mG6PC1 from Sf9 membranes using a three-step approach incorporating immobilized metal affinity chromatography (IMAC), proteolytic cleavage of the EGFP fusion, and size-exclusion chromatography (SEC). An octa-Histidine tag positioned at the C-terminus of EGFP facilitated the isolation of the fusion construct *via* Ni^2+^-NTA coordination, and thrombin protease was used to cleave off EGFP prior to the final gel filtration step. From this process, we were able to obtain milligram quantities of mG6PC1 (see Experimental procedures for more details).

**Figure 3:**
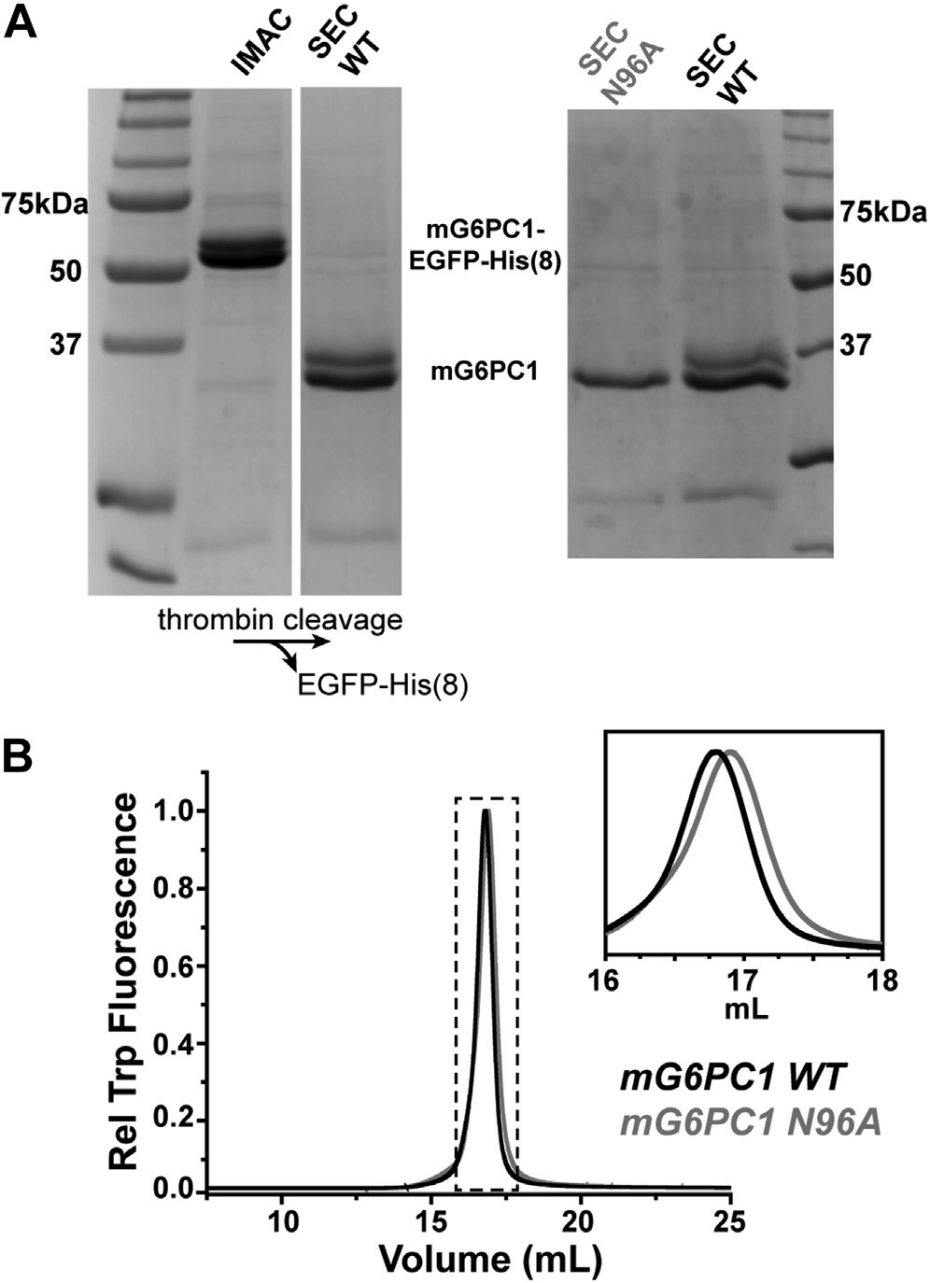
**Purification of mG6PC1 from Sf9 cell membranes.***A*, SDS-PAGE of WT mG6PC1 shows two bands consistent with a glycosylated and nonglycosylated product ∼40 kDa in size. Accordingly, the purified N96A variant, which prevents glycosylation, is a single band. The two image panels on the *left* come from the same gel, whereas the image panel on the *right* comes from a different preparation. *B*, FSEC traces of fully purified mG6PC1-WT and -N96A highlight the subtle shift to lower hydrodynamic radius of the N96A variant. FSEC, fluorescence detection size-exclusion chromatography; IMAC, immobilized metal affinity chromatography; SEC, size exclusion chromatography.

mG6PC1-WT appeared as a doublet on SDS-PAGE gels (Fig. 3*A*), which likely arose from incomplete *N*-linked glycosylation of Asn96 (36). Treatment of purified mG6PC1-WT with Peptide-*N*-Glycosidase F (PNGase F) collapsed the doublet into a single band (Fig. S4). Consistent with this result, SDS-PAGE of purified Ala variant (N96A) from Sf9 cells (Fig. 3*A*) displayed a single band corresponding to the lowest-molecular-weight species of the WT. Similarly, Western blot of N96A expressed in INS-1 832/13, a cell line that has been used to study mG6PC1 (37), revealed a single band in comparison to the doublet observed with mG6PC1-WT (Fig. S4). Densitometry analysis of multiple independent preparations suggested that approximately 40% of mG6PC1-WT was glycosylated when isolated from Sf9 cells. Importantly, purified mG6PC1-WT and -N96A displayed homogeneous preparative SEC and analytical FSEC traces (Figs. 3*B* and S5), which indicated that the N96A variant was not associated with protein misfolding.

**Figure 4:**
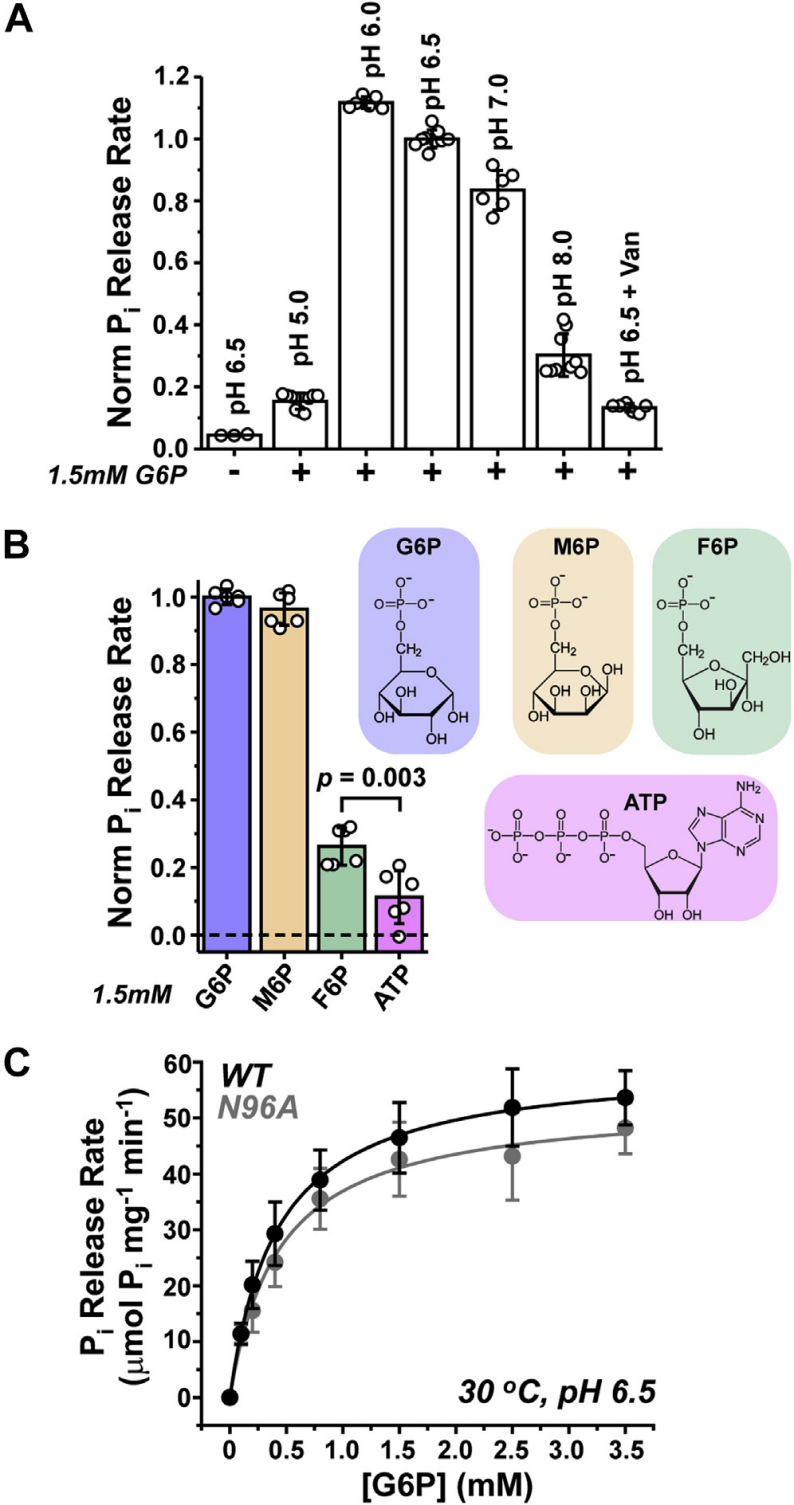
**Catalytic profile of purified mG6PC1.***A*, G6P hydrolysis is most active between pH 6.0 and 7.0, and mostly inhibited in the presence of 1 mM vanadate. *B*, whereas G6P and M6P are hydrolyzed similarly at pH 6.5 and 30 °C, hydrolysis of F6P and ATP are attenuated strongly. An unpaired *t* test between relative P_i_ release rates from F6P (0.26 ± 0.05) and ATP (0.11 ± 0.08) was used to compare the means and determine the *p* value shown. *C*, kinetic analysis of mG6PC1-WT and -N96A show similar rates of P_i_ release from G6P hydrolysis. *Solid lines* are nonlinear least squares fits of the velocity data assuming a Michaelis–Menten model. F6P, fructose-6-phosphate; G6P, glucose-6-phosphate; M6P, mannose-6-phosphate.

We screened the catalytic profile of fully purified mG6PC1 to characterize the pH sensitivity, identify preferred substrates, and measure G6P turnover kinetics. In these assays, we found that mG6PC1 activity recapitulated the general biochemical properties previously observed within intact microsomes (27, 29, 31, 38). For example, P_i_ release from G6P hydrolysis was strongly pH-dependent (Fig. 4*A*). The highest activity was observed within a narrow band of one pH unit from pH 6.0 to 7.0, consistent with the proposed mechanism of conserved Arg and His side chains mediating G6P hydrolysis (22, 24, 39). Vanadate, which is predicted to bind competitively to the orthosteric substrate-binding site forming a stable trigonal bipyramidal structure (38, 40), potently inhibited P_i_ release (Fig. 4*A*).

Although G6P is known to be the endogenous substrate of G6PC1, the enzyme has been observed to catalyze hydrolysis of other sugar phosphates *in vitro* (3, 9, 33). We investigated the phosphohydrolase activity of mG6PC1 on a select panel of phosphate esters (Fig. 4*B*). Since it is not transported by the G6P transporter (SLC37A4), mannose-6-phosphate (M6P) has been used to measure the latent G6PC1 activity within permeabilized microsomes (3, 6, 41, 42). Figure 4*B* shows that this G6P stereoisomer was hydrolyzed at a similar rate as the native substrate, as expected (3). In contrast, the P_i_ release rate of fructose-6-phosphate (F6P), a G6P precursor in the gluconeogenesis pathway, was ∼30% of that observed for G6P and M6P. Nevertheless, catalysis of F6P exceeded that of ATP, which is not considered a natural substrate for the phosphohydrolase function of G6PC1 but rather may operate as a competitive inhibitor (33).

Despite the agreement with studies in microsomes, Michaelis–Menten modeling of the [G6P]-dependent changes in the P_i_ release rate of purified mG6PC1 revealed unique kinetic parameters (Fig. 4*C* and Table 1). The *K*_m_ was substantially lower than previous measurements of mouse, rat, or human G6PC1 in intact microsomes (*K*_m_ = 2–3 mM) (3, 34, 38, 43, 44). Strikingly, *V*_max_ was 2 to 3 orders of magnitude higher than that obtained from crude enzyme preparations (34, 38, 43, 44, 45). In accord with limited hydrolysis of F6P (Fig. 4*B*), catalytic efficiency (*k*_cat_/*K*_m_) was >10-fold higher for G6P than F6P (Fig. S6). Although a subtle reduction in *V*_max_ for N96A relative to WT was observed (∼10%, Fig. 4*C* and Table 1), an unpaired *t* test suggested that the mean difference of the current dataset is statistically not significant by conventional criteria (*p* = 0.2244; 95% confidence interval of the mean difference: −5.5481 to 18.5281).

**Table 1:** Kinetic parameters of purified mG6PC1-WT and -N96A

mG6PC1	Micelle^a^	*K*_M_ (mM)^b^	*V*_max_ (μmol P_i_ mg^−1^ min^−1^)^b^	*k*_cat_ (s^−1^)	*k*_cat_/*K*_M_ (M^−1^ s^−1^)
WT	LMNG	0.431 ± 0.08	60.14 ± 5.39	41	9.6 × 10^4^
	LMNG + CHS	0.474 ± 0.04	47.05 ± 1.94	32	6.8 × 10^4^
	LMNG + POPC	0.438 ± 0.04	58.46 ± 2.36	40	9.2 × 10^4^
N96A	LMNG	0.454 ± 0.08	53.65 ± 7.10	37	8.2 × 10^4^

Data reported is mean ± standard deviation.

a 0.2 mM LMNG with or without 0.02 mM CHS or 0.02 mM POPC.

b n = 3 to 5 replicates collected at pH 6.5 and 30 °C.

### Lipids enhance catalytic and structural stability of mouse G6PC1

The stable isolation of mG6PC1 in a functional form afforded an opportunity to investigate the role of lipids on biophysical and biochemical properties. Among the noted differences from the plasma membrane, the lipid composition of ER membranes is enriched particularly in phosphatidylcholine (>50 mol%) yet depleted in cholesterol (∼5 mol%) (46, 47, 48, 49). Therefore, we explored catalysis and stability of mG6PC1 when purified into LMNG micelles supplemented with the model lipid bilayer species 1-palmitoyl-2-oleoyl-glycero-3-phosphocholine (POPC) or cholesteryl hemisuccinate (CHS), a cholesterol analog with higher aqueous solubility (50). While POPC and CHS are typical lipid and sterol representatives in biophysical and structural studies (51, 52, 53, 54, 55, 56), POPC has been used specifically as an ER-mimicking lipid (57).

**Figure 5:**
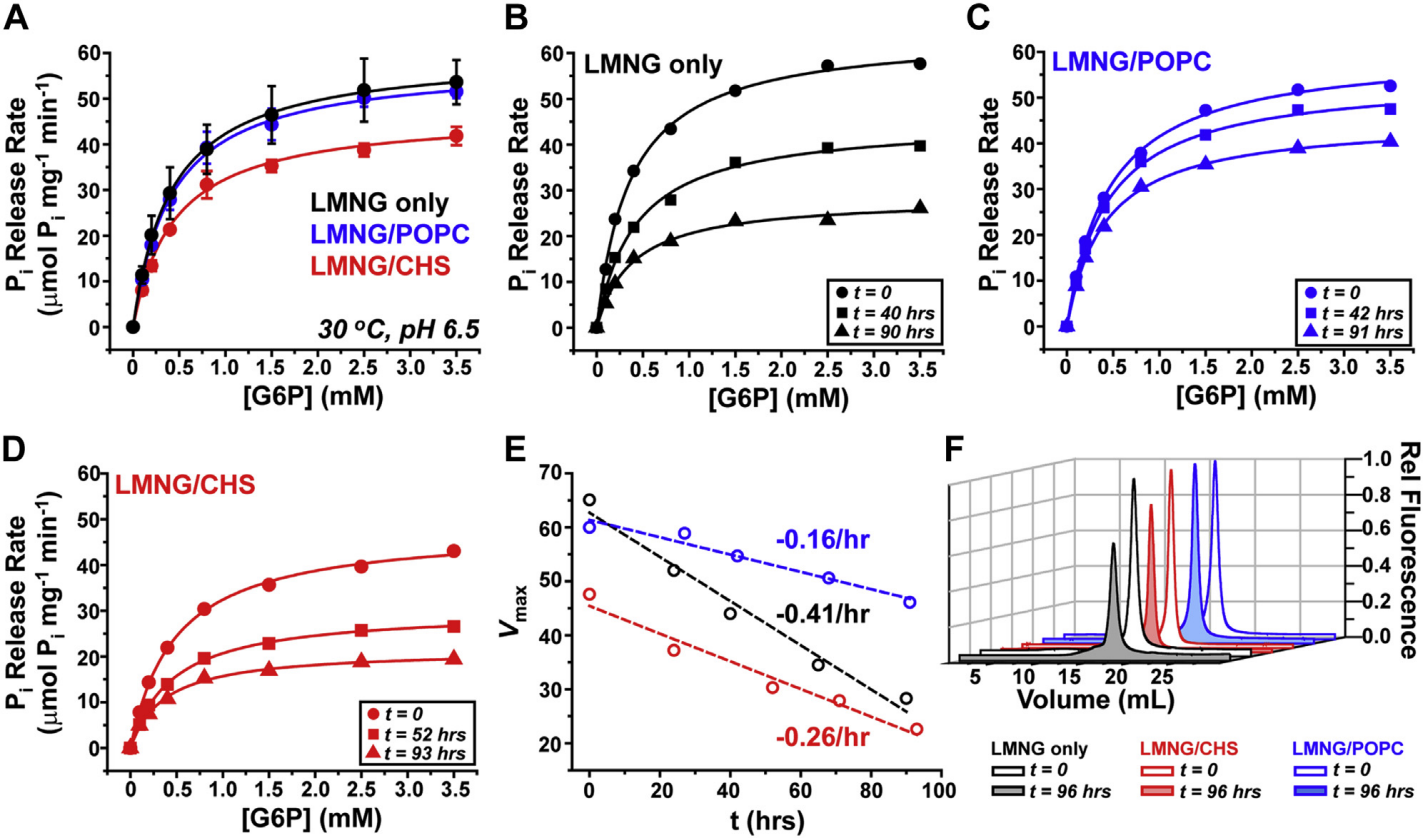
**Stabilization of mG6PC1 catalysis by lipids.***A*, saturation kinetics of P_i_ release indicates a reduction in *V*_max_ in the presence of CHS. *B*–*D*, P_i_ release curves in the presence of POPC or CHS are more clustered over time relative to LMNG alone. Each curve represents the average of two independent measurements acquired at 30 °C and pH 6.5 buffer. *E*, the change in *V*_max_ over time reveals a reduction in catalytic decay rate in the presence of POPC (*blue*) or CHS (*red*). *Dashed lines* are linear regressions of the data, and the slopes correspond to the loss of turnover velocity per hour. *F*, FSEC of mG6PC1 reports a loss of soluble enzyme after 96 h in LMNG micelles (*black lines*) as indicated by a decrease in fluorescence intensity. The presence of CHS (*red lines*) or POPC (*blue lines*) mitigates this loss over the same time period. CHS, cholesteryl hemisuccinate; FSEC, fluorescence detection size-exclusion chromatography; LMNG, lauryl maltose neopentyl glycol; POPC, 1-palmitoyl-2-oleoyl-glycero-3-phosphocholine.

A comparison of the substrate-dependent kinetics of mG6PC1-WT purified in the three micellar conditions is shown in Figure 5*A*, and the fit parameters are recorded in Table 1. Whereas the *K*_m_ remained largely unchanged in all conditions tested (mean = 0.45 ± 0.05 mM, n = 13), *k*_cat_ was depressed approximately 20% in the presence of CHS. In contrast, no substantial changes in *k*_cat_ were observed in the presence of POPC, suggesting that the cholesterol derivative specifically reduced G6P turnover velocity. Despite this result, the presence of either POPC or CHS increased the catalytic stability of mG6PC1. To capture this behavior, the P_i_ release rate was measured over a 4-day period for each condition. Representative curves derived from duplicate measurements are shown in Figure 5, *B*–*D*. Relative to the time course in LMNG alone (Fig. 5*B*), P_i_ release curves clustered more tightly in the presence of either POPC (Fig. 5*C*) or CHS (Fig. 5*D*). Indeed, a plot of *V*_max_ as a function time revealed that inclusion of POPC or CHS reduced the rate of catalytic decay approximately two- to threefold relative to LMNG alone (Fig. 5*E*).

Notably, the decline of phosphohydrolase activity was associated with the apparent depletion of homogeneous mG6PC1 over the time course of the experiment. As shown in Figure 5*F*, the FSEC elution peak of mG6PC1 indicated a ∼40% loss of enzyme after 96 h (black traces), which was abrogated substantially by the presence of either CHS (∼20% loss, red traces) or POPC (no apparent loss, blue traces). This result suggested that the increased catalytic stability was in part the consequence of elevated structural stability conferred by doping LMNG proteomicelles with POPC or CHS.

**Figure 6:**
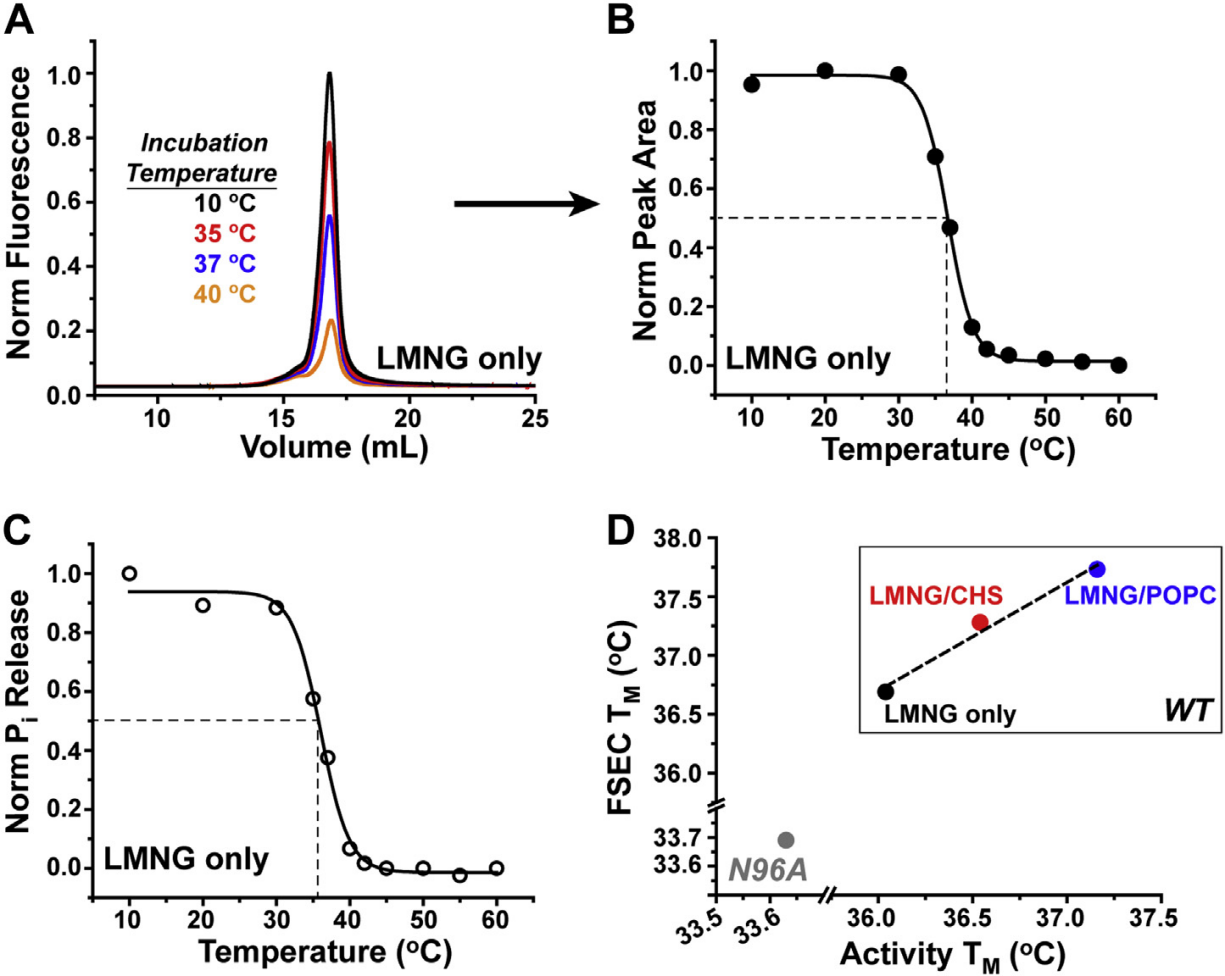
**Lipids increase thermostability of mG6PC1.***A*, FSEC illustrates the temperature-dependent loss of mG6PC1 from the soluble fraction in LMNG micelles. Fitting of the decay in peak area as a function of temperature yields the T_m_ (*B*). The temperature-dependent decay of phosphohydrolase activity measured at 30 °C in pH 6.5 buffer reports a similar T_m_ (*C*). *D*, supplementing LMNG micelles (*black*) with CHS (*red*) or POPC (*blue*) supports an increase in thermostability, rationalizing the reduced catalytic decay rate in the presence of lipids shown in Figure 5. In contrast, the N96A variant (*gray*) reported a reduction in thermostability. The scales of the x- and y-axes are broken in order to map the T_m_ of mG6PC1-WT and -N96A onto the same plot. The *dashed line* in (*D*) is a linear regression of the WT data (slope = 0.92). CHS, cholesteryl hemisuccinate; FSEC, fluorescence detection size-exclusion chromatography; LMNG, lauryl maltose neopentyl glycol; POPC, 1-palmitoyl-2-oleoyl-glycero-3-phosphocholine.

In order to test this hypothesis, we characterized the thermostability of mG6PC1 by explicit measurement of the melting temperature (T_m_) of mG6PC1 as assessed by FSEC analysis (58) and G6P hydrolysis. In these complementary assays, mG6PC1 purified in the presence or absence of CHS or POPC was incubated at defined temperatures for 10 min and then subjected to FSEC analysis (Trp fluorescence, Fig. 6, *A* and *B*) and activity measurements (P_i_ release, Fig. 6*C*). The temperature-induced depletion of soluble, active enzyme was fit directly to determine T_m_, the temperature at which 50% of the enzyme signal remained. Shown as a representative dataset, a strong correspondence in the reported T_m_ for mG6PC1-WT purified in LMNG micelles was obtained from FSEC (Fig. 6*B*) and G6P hydrolysis (Fig. 6*C*). Consistent with the hypothesis that lipids enhanced catalytic and structural stability, supplementing LMNG micelles with either CHS or POPC supported an increase in the thermostability of mG6PC1-WT characterized by a linear correlation between the T_m_ derived from FSEC and G6P hydrolysis (Figs. 6*D* and S7). In contrast to the WT, the thermostability of the N96A variant (purified into LMNG micelles only) was decreased ∼10% (Figs. 6*D* and S7), suggesting compromised structural and catalytic stability of the mutant.

## Discussion

The unique insights into the enzymology of G6PC1 presented here were made possible by resolving the core issue of catalytic instability that arises primarily from sensitivity to the solubilizing detergent (3, 28). The FSEC and activity screens identified LMNG as a detergent that solubilizes homogeneous and functional G6PC1. As shown previously for a variety of membrane proteins (59), solubilization of either hG6PC1 or mG6PC1 with LMNG markedly improved structural stability and functional activity relative to the widely-used β-C12M. This critical discovery facilitated purification of stable G6PC1 from a heterologous host while retaining definitive catalytic features. Accordingly, these exceptional results establish new methodological and experimental paradigms for the biophysical and biochemical analysis of G6PC1.

Due to the higher intrinsic stability relative to the human ortholog, we took advantage of mG6PC1 as an archetype for *in vitro* kinetic experiments. In addition to confirming formerly observed functional properties (*i.e.*, pH dependence, substrate specificity, and vanadate inhibition), kinetic analysis of purified mG6PC1 uncovered novel catalytic parameters buttressed by the highest specific activity reported to date (BRENDA:EC3.1.3.9). The increased apparent affinity for G6P (*K*_m_) and considerably elevated catalytic capacity (*V*_max_ and *k*_cat_) relative to prior reports are likely reflective of a uniform solvent environment unlike that encountered with crude microsome preparations combined with the ability to more readily determine the concentration of purified enzyme. Permeabilization of microsomes with detergents has been shown to substantially decrease *K*_m_ (3, 9, 33). This observation suggests that detergent treatment of microsomes increases access to the G6PC1 active site, reduces substrate interactions with other binding partners, releases endogenous enzyme modulators, or a combination of these. Unencumbered by these variables, our kinetic measurements of mG6PC1 activity directly determined the catalytic rate constant and turnover velocity under stable biochemical conditions for the first time.

In the physiological context of the ER, access of G6P to the G6PC1 active site in the ER lumen requires substrate shuttling from the cytosol by the endogenous SLC37A4 transporter (5, 6). Studies have shown clearly that perturbing mutations in either component disrupt glucose homeostasis (60, 61), highlighting the coordinated functional interaction between the transporter and catalytic subunit to mediate glucose production. We note that the phosphohydrolase activity of G6PC1 reported here exceeds the estimated G6P uptake rates by SLC37A4 crudely reconstituted into proteoliposomes by three orders of magnitude (4, 62). This observation might suggest that glucose production may be limited principally by transport of G6P from the cytosol (3, 61). However, overexpression of either G6PC1 or SLC37A4 elevates hepatic glucose production (14, 63, 64), indicating that the tight coupling of G6PC1 with SLC37A4 determines the actual rate of glucose production *in vivo* (65). Therefore, the relationship of transport to G6P hydrolysis requires further investigation with fully purified components to delineate the mechanistic details of coupling.

Similar to the human ortholog (36), mG6PC1 retains a single conserved glycosylation site at Asn96 (Fig. 3). In a previous study of hG6PC1-N96A, enzyme synthesis and degradation were unaltered relative to the WT despite a decrease in phosphohydrolase activity (36). However, oligosaccharide modification of glycoproteins has been shown to facilitate protein folding and improve stability (66, 67, 68). The purification of homogeneous and catalytically active mG6PC1-N96A indicated that glycosylation is not critical for folding. However, the thermostability analysis (Fig. 6) implied that the N96A mutation reduced both structural and catalytic stability relative to WT. Although this observation predicts that glycosylation stabilizes mG6PC1-WT, the blunt nature of the N96A mutation may induce marginal instability of the enzyme intrinsically.

Remarkably, supplementing LMNG micelles with either POPC or the cholesterol analog CHS enhanced the structural and catalytic stability of purified mG6PC1. This observation argues for specific lipid–protein interactions that affect enzyme structure and activity and warrants further investigation into the role(s) of other lipid species. For example, phosphatidylinositol, which is enriched also in the ER membrane (46), and the derivative phosphoinositides have been suggested to function as competitive inhibitors of G6PC1 *in vitro* (69). In addition to its stabilizing properties, we found that CHS also reduced *k*_cat_, indicating a potential role for cholesterol-dependent allosteric modulation. Similar to previous estimates (33, 70, 71), Arrhenius analysis revealed a mean activation energy of 10.4 ± 0.8 kcal/mol (n = 3) across all micellar conditions (Fig. S8), suggesting that the relative stability of the high-energy transition state is not substantially changed in the presence of either POPC or CHS. Therefore, CHS may reduce the P_i_ release rate by altering enzyme conformation or destabilizing the enzyme–substrate complex. We note that while CHS has been proposed to be a reasonable cholesterol mimic in bilayers and has been applied in structural and mechanistic studies of membrane proteins (50, 51, 72, 73), it is not clear if the succinate moiety alters protein–sterol interactions. Therefore, subsequent studies examining the role of cholesterol in the context of proteoliposomes or nanodiscs that mimic ER lipid composition will be necessary to clarify this point.

In combination with the recent computational model (26), the technical advancements showcased in this study set the stage for mechanistic interrogation of G6PC1 and known pathogenic mutations in unprecedented detail. More than 100 genetic variants of G6PC1 have been associated with GSD type 1a, the vast majority of which are missense mutations that are distributed throughout the primary sequence (19, 74, 75, 76). We expect that employing the methodologies and assays described here will add a new dimension to the molecular understanding of these debilitating mutations in G6PC1 that reside at the intersection of enzyme expression, folding, stability, and catalysis.

## Experimental procedures

### Genes and construct design

For screening in mammalian cells, WT mouse (accession number NM_008061) and human (accession number BC130478) G6PC1 were cloned as EGFP fusion proteins into the pJPA5 MOD expression vector. The EGFP cassette encoding a 5′ thrombin recognition sequence and 3′ octa-His (His_8_) tag was ligated in frame with the G6PC1 sequence. The parent vector pJPA5 was a generous gift from Dr David Jacobson (Vanderbilt University). This vector was modified by insertion of a polylinker containing the following restriction enzyme sites 3′ of the CMV promoter and 5′ untranslated region sequence: EcoR I, HinD III, Bgl II, BamH I, EcoR V, Xho I, and Pme I creating the plasmid pJPA5 MOD. Mouse and human G6PC1 EGFP fusion proteins were inserted into pJPA5 MOD with a leading Kozak sequence using the EcoR I and Xho I restriction sites. Site-directed mutagenesis using the Quikchange II kit (Agilent Technologies) was used to generate the N96A variant in mG6PC1-EGFP. DNA sequencing was used to verify the presence of the correct codon change and the absence of secondary mutations in the open reading frame. For insect cell expression, the WT and N96A mG6PC1-EGFP fusion cassettes were shuttled from pJPA5 MOD into pFastBac1 using the EcoR I and Xho I restriction sites and confirmed by DNA sequencing.

### Screening of mG6PC1-EGFP and hG6PC1-EGFP in mammalian cells

#### Transfection

Adherent HEK293S GnTI^−^ (*N*-acetylglucosaminyl-transferase I—negative; ATCC CRL-3022) cells cultured in DMEM medium supplemented with 10% FBS were seeded in a 6-well plate (Costar) and transfected at ∼50 to 75% confluence with 2 μg/well of plasmid DNA encoding either mG6PC1-EGFP or hG6PC1-EGFP in the pJPA5 MOD expression vector complexed with Lipofectamine 2000 reagent (Invitrogen) following the manufacturer’s protocol. The cells were incubated for 48 h at 37 °C under 7% CO_2_. Expression was confirmed by visualizing cell epifluorescence on a Zeiss Axio Zoom.V16 fluorescence stereo microscope. For each construct, ten wells total were transfected per experimental iteration for screening by FSEC and phosphohydrolase activity. At harvest, the media was removed by aspiration and each well gently washed with 1 ml of 50 mM Tris pH 8.0, 50 mM NaCl, 0.5 mM EDTA, 10% (v/v) glycerol buffer and transferred to a 15-ml conical on ice. A portion of the resuspended cells was dedicated for FSEC analysis, while the remainder was allocated for activity (P_i_ release) assays (Fig. 1).

#### FSEC

For each construct, the washed cells were distributed evenly into two 1.5 ml Eppendorf tubes and centrifuged at 2300 rcf for 4 min. The cell pellets were solubilized in 300 μl buffer composed of 50 mM Tris-HCl pH 8.0, 150 mM NaCl, 1 mM EDTA supplemented with either 5 mM (0.5% w/v) LMNG or 40 mM (2% w/v) β-C12M, and 5 mM PMSF while nutating for 1 h at 4 °C. Insoluble material was removed by ultracentrifugation at 105,000*g* for 20 min. One hundred microliters of supernatant containing solubilized enzyme was injected at 0.5 ml/min onto a Superose6 Increase 10/300 GL column equilibrated in 50 mM Tris-HCl pH 8.0, 150 mM NaCl, 1 mM EDTA, and 0.01% (w/v) LMNG buffer. The column was attached to an Agilent 1260 Infinity II chromatography system equipped with a fluorescence detector and a temperature-controlled multisampler. Sample elution profiles monitored EGFP fluorescence (Ex 475 nm, Em 515 nm) with an instrument gain of 15 and data collection frequency of 9.3 Hz (1 s response time). A second 100 μl injection of the same sample was performed to ascertain stability following 48 h incubation on ice.

#### P_i_ release measurements

For each construct, the remainder of the washed cells were distributed evenly into two 1.5 ml Eppendorf tubes and centrifuged at 2300 rcf for 4 min. The cell pellets were solubilized in 500 μl hydrolysis reaction buffer composed of 50 mM Tris/Mes pH 6.5, 50 mM NaCl, 1 mM EDTA supplemented with either 5 mM (0.5% w/v) LMNG or 40 mM (2% w/v) β-C12M, and 5 mM PMSF nutating for 1 h at 4 °C. Insoluble material was removed by ultracentrifugation at 105,000*g* for 20 min.

To initiate hydrolysis, 20 μl of supernatant containing solubilized enzyme was diluted into 150 μl hydrolysis reaction buffer supplemented with either 1 mM β-C12M or 0.2 mM LMNG in the presence or absence of 1.5 mM G6P while on ice, then transferred to a 30 °C water bath for 5 min. Reactions were quenched with 150 μl of 12% (w/v) SDS and vortexed. The reactions were performed in triplicate per experimental iteration (n = 3) and repeated after 24 h and 48 h incubation on ice.

To determine the amount of P_i_ released, we adapted a previous protocol used for the quantification of P_i_ released from ATP hydrolysis induced by ABC transporter activity (77). Briefly, the quenched reactions were mixed with 300 μl P_i_ chelator (1% ammonium molybdate +6% ascorbic acid in H_2_O and 1.2 M HCl) for 5 min. Subsequently, 450 μl of developing solution composed of 2% (w/w) sodium citrate, 2% (w/w) sodium meta-arsenite, and 0.35 M glacial acetic acid was added to each sample and mixed. After incubation at room temperature for 20 min, the sample absorbance at 850 nm was read on a BioTek Synergy H4 microplate reader. The correspondence of 850 nm to the total nmol of P_i_ released was determined from a P_i_ standard curve. To account for differences in expression level across multiple experimental iterations, the nmol of P_i_ released was divided by the FSEC peak area and then normalized to the activity of mG6PC1-EGFP solubilized in LMNG. The statistical error was propagated accordingly.

### Screening of mG6PC1-EGFP in Sf9 cells

#### Recombinant baculovirus production

WT mG6PC1-EGFP in the pFastBac1 vector was transformed into DH10Bac competent cells for site-specific transposition of the expression cassette into bacmid. Purified bacmid was transfected into adherent Sf9 cells seeded at 1 × 10^6^ cells per well of a 6-well dish using the Cellfectin II reagent. Cells were incubated for 5 days at 27 °C prior to harvesting and filtering the media that contained P1 baculovirus. To amplify the virus, P1 was diluted 1000-fold into Sf9 suspension cells at 1 × 10^6^ cells/ml growing in serum free Sf-900-II SFM media (Gibco) and incubated for 4 days at 27 °C with shaking (130 rpm). The cells were removed by centrifugation, and the supernatant containing P2 baculovirus was filter sterilized (0.2 μm). P1 and P2 baculovirus was stored at 4 °C protected from light and supplemented with 2% FBS. The titer of P2 virus was determined by end-point dilution assay in Sf9 cells similar to that previously described (78).

#### Titration of baculovirus

Five flasks containing 50 ml of 2.5 × 10^6^ cells/ml Sf9 cells cultured in suspension were transduced with five independent concentrations of titered baculovirus carrying the mG6PC1-EGFP fusion. The absolute multiplicity of infection (MOI) for each flask was in the approximate following ranges: 0.5 to 0.2 (flask 1), 0.25 to 0.1 (flask 2), 0.15 to 0.06 (flask 3), 0.05 to 0.02 (flask 4), and 0.005 to 0.002 (flask 5). Relative to flask 1, these ranges correspond to a relative MOI (Rel MOI) of 1, 0.5, 0.3, 0.1, and 0.01, respectively. The Rel MOI was used in Figure 2 and Fig. S3 to account for variability in the absolute baculovirus titer across multiple preparations of P2 virus used for the titration experiments, thus simplifying the analysis. The cells were incubated at 27 °C with shaking (130 rpm) for 24 h prior to harvesting the cells by centrifugation. Expression was confirmed by visualizing cell epifluorescence on a Zeiss Axio Zoom.V16 fluorescence stereo microscope. Under these conditions, the expression of mG6PC1-EGFP was linear as shown in Figure 2 and Fig. S3. However, deviation from linearity was observed when incubation continued for 30 h or more (data not shown).

#### FSEC

One milliliter of cells was pelleted by centrifugation and then solubilized in 400 μl composed of 50 mM Tris-HCl pH 8.0, 150 mM NaCl, 1 mM EDTA supplemented with 5 mM (0.5% w/v) LMNG, and 5 mM PMSF while nutating for 1 h at 4 °C. Insoluble material was removed by ultracentrifugation at 105,000*g* for 20 min. One hundred microliters of supernatant containing the solubilized mG6PC1-EGFP was injected onto the Superose6 Increase 10/300 GL column as described above. The relative concentration of mG6PC1-EGFP was estimated from integration of the elution peak.

#### P_i_ release measurements

Twenty milliliters of cells from each flask was pelleted by centrifugation and resuspended in 12 ml of lysis buffer (50 mM Tris pH 8.0, 50 mM NaCl, 0.5 mM EDTA, 10% (v/v) glycerol) supplemented with 5 mM PMSF. The cells were disrupted by micro-tip sonication with 120 to 1 s pulses (10 s off duty, 20% amplitude) while on ice. The lysate was centrifuged at 8000 rcf for 10 min, and then the supernatant subjected to ultracentrifugation at 105,000 rcf for 30 min to isolate the membrane fraction. The membranes were resuspended in hydrolysis reaction buffer (50 mM Tris/Mes pH 6.5, 50 mM NaCl) to 80 mg/ml. Next, 125 μl of this membrane suspension was diluted to 500 μl in hydrolysis reaction buffer supplemented with 5 mM (0.5% w/v) LMNG (20 mg/ml membranes). After 1 h of nutation at 4 °C, insoluble material was removed by ultracentrifugation at 105,000*g* for 20 min. Seventy-five microliters of supernatant containing the solubilized enzyme was mixed with 75 μl of hydrolysis reaction buffer in the presence or absence of 1.5 mM G6P while on ice, then transferred to a 30 °C water bath for 5 min. Reactions were quenched with 150 μl 12% (w/v) SDS and vortexed. Reactions were performed in triplicate. The samples were developed for P_i_ content as described above. The P_i_ released shown in Figure 2*C* was normalized relative to the activity in the Rel MOI = 1 cells.

### Expression of mG6PC1 in 832/13 cells and Western blot analysis

Rat islet-derived 832/13 cells (79) were passaged as subconfluent cultures in RPMI medium supplemented with 10% (vol/vol) fetal bovine serum, 0.05 mM β-mercaptoethanol, 100 U/ml penicillin, and 100 mg/ml streptomycin. Plasmids (3 μg) encoding mG6PC1-WT and -N96A were transfected into semiconfluent 832/13 cells in 3.5 cm diameter dishes using the lipofectamine reagent (InVitrogen) as previously described (80). Cells were then incubated for 18 to 20 h in serum-containing medium before harvesting using trypsin, pelleting at 3500*g* for 1 min at room temperature, washing in PBS, and resuspending in 50 mM Tris, pH 8.0, 150 mM NaCl, 5.8 mM PMSF, and 1% NP-40. The Pierce BCA Protein Assay kit (Thermo Fisher Scientific) was used for protein quantitation. Cell extract (5 μg) was electrophoresed on 10% SDS–polyacrylamide gels and the proteins transferred to PVDF membrane (PerkinElmer). Protein expression was then determined by immunoblotting using the following antibodies: (1) a primary anti-beta actin monoclonal antibody (1:10,000, Sigma) with a secondary anti-mouse horseradish peroxidase (HRP) secondary antibody (1:10,000, Promega), or (2) a conjugated mouse monoclonal anti-V5-horseradish peroxidase (HRP) antibody (1:100–1:5000, InVitrogen), as stated. HRP activity was assayed using the PierceECL reagent (Thermo Fisher Scientific), and beta actin expression was used as a loading control.

### Expression and purification of mG6PC1 from Sf9 membranes

Based on preliminary screens, optimal mG6PC1-EGFP expression was obtained from Sf9 cells cultured in suspension (2.5–3 × 10^6^ cells/ml) when transduced with baculovirus at an absolute MOI of 3 to 5 for 72 h at 27 °C with shaking (130 rpm). The cells were processed on ice by sonication (60 cycles of 10 s on/off, 40% amplitude) in lysis buffer supplemented with a protease inhibitor cocktail (l0 μg/ml leupeptin, l0 μg/ml pepstatin, 5 μg/ml chymostatin, 1 mM benzamidine, and 1 mM PMSF). Cell debris was removed by centrifugation at 10,000 rcf for 20 min, followed by isolation of the membrane fraction *via* ultracentrifugation at 185,000 rcf for 1.5 h. Four to five grams of membranes was homogenized in IMAC-binding buffer (50 mM Tris pH 8, 100 mM NaCl, 10% (v/v) glycerol) and solubilized with 0.06 g LMNG/g of membrane for 1 h on ice. The final detergent concentration was 5 mM (0.5% w/v). Insoluble material was removed by ultracentrifugation at 185,000 rcf for 50 min.

The supernatant containing the solubilized enzyme was filtered with a 0.45 μm syringe filter and mixed with 2 ml (bed volume) of Ni^2+^-NTA Superflow (Qiagen) equilibrated in binding buffer supplemented with 25 mM imidazole for 4 h at 4 °C with gentle shaking. The resin was washed with ten column volumes of binding buffer containing 50 mM imidazole and 0.2 mM LMNG (0.02% w/v). The sample was released from the resin by application of 300 mM imidazole. The concentration of mG6PC1-EGFP was estimated from absorbance at 280 nm assuming a 112,355 M^−1^ cm^−1^ molar extinction coefficient calculated from the protein sequence (81). On average, yields were estimated to be greater than 0.7 mg/g of membrane following affinity chromatography. The sample was treated with thrombin protease (Sigma) using a 0.04 NIH units/μg of mG6PC1-EGFP ratio for 16 h at 15 °C. After ultracentrifugation to remove any precipitation formed during protease treatment, pure mG6PC1 was pooled from appropriate fractions obtained by SEC on a Superose6 Increase 10/300 GL column equilibrated with 50 mM Tris pH 7.5, 150 mM NaCl, 0.2 mM LMNG. The column was attached to a Bio-Rad Bio-Logic DuoFlow chromatography system equipped with a UV detector and fraction collector operating at 4 °C. For preparations including lipids, the initial detergent solubilization included 0.82 mM (0.05% w/v) CHS or 0.5 mM (0.04% w/v) POPC. During affinity and SEC, the lipid concentrations were reduced to 0.02 mM (0.0012% w/v, 5.7 mol%) CHS or 0.02 mM (0.0016% w/v, 7 mol%) POPC. The LMNG molar concentration was ten times the lipid concentration. Sample purity was assessed by SDS-PAGE using a 13% acrylamide gel (Fig. 3). Chromatographic behavior was assayed with FSEC by injecting 7 μg of the purified enzyme onto the Superose6 Increase 10/300 GL column and monitoring fluorescence (Ex 280 nm, Em 320 nm; gain = 10).

### Phosphohydrolase assays of purified mG6PC1

Catalytic parameters of P_i_ release were derived from titration of 0.1 μg (∼2.4 pmol) mG6PC1 with G6P or F6P (0–3.5 mM) incubated at 30 °C for 1 min in pH 6.5 hydrolysis reaction buffer supplemented with 0.2 mM LMNG. A linear [G6P]-dependent curve obtained at 0 °C reflecting the free P_i_ background carried over from the G6P stock was subtracted from the curve acquired at 30 °C. The resulting hyperbola was fit with a Michaelis–Menten model. Measurements of catalytic stability in the presence or absence of 0.02 mM CHS or POPC were obtained from duplicate curves obtained at the indicated time points (Fig. 5) over ∼96 h of the sample stored on ice. The activation energy (*E*_a_) was estimated from the average of 2 to 3 independent curves measured at 20, 25, and 30 °C for all three micellar conditions. G6P hydrolysis at 15 °C deviated from linearity in the Arrhenius plot and was not included in the final *E*_a_ calculation.

The pH dependent profile of P_i_ release (Fig. 4) was determined by diluting purified mG6PC1 ∼300-fold into appropriate buffers in the presence of 1.5 mM G6P for 1 min at 30 °C. Orthovanadate (Na^+^ salt) inhibition was assayed at 1 mM. Measurements were performed in triplicate with 2 to 3 independent experimental iterations. The estimated P_i_ release rate was normalized relative to the rate observed at pH 6.5. Likewise, the estimated P_i_ release rate from various substrates was determined by hydrolysis of 1.5 mM substrate for 1 min at 30 °C in pH 6.5 buffer. Measurements were performed in triplicate with two independent measurements. The estimated P_i_ release rate was normalized relative to the rate observed with G6P.

### Thermostability analysis

Purified mG6PC1 (WT or N96A) was diluted to 0.1 mg/ml in 50 mM Tris pH 7.5, 150 mM NaCl, 0.2 mM LMNG buffer in the absence and presence of 0.02 mM lipids and transferred to standard PCR tubes for incubation in a thermocycler at defined temperatures. For each temperature, 100 μl of sample was heated for 10 min and then transferred immediately to ice. The samples were subjected to ultracentrifugation at 105,000*g* for 20 min to remove precipitated material prior to injecting 70 μl of supernatant onto a Superose6 Increase 10/300 GL column equilibrated in the same buffer. Elution from the column was monitored by Trp fluorescence. Another 10 μl of heat-treated supernatant was diluted 150-fold into hydrolysis reaction buffer for measurement of phosphohydrolase activity toward 1.5 mM G6P at 30 °C for 1 min. The FSEC peak area and the phosphohydrolase activity at each temperature were normalized to the value at 10 °C and plotted as a function of increasing temperature for fitting with a dose–response curve to determine the melting temperature (T_m_) as shown in Figure 6.

## Data availability

All data described have been included in the manuscript.

## Supporting information

This article contains supporting information.

## Conflict of interest

The authors declare that they have no conflicts of interest with the contents of this article.
