# Supplementary material for: Biophysical and functional properties of purified glucose-6-phosphatase catalytic subunit 1
Source: J Biol Chem. 2021 Dec 21;298(1):101520. doi: 10.1016/j.jbc.2021.101520 (PMC8753184; doi:10.1016/j.jbc.2021.101520)
Supplement: Supplemental Figures S1–S8 [file mmc1.pdf]

Supporting Information for

Biophysical and functional properties of the purified catalytic subunit of glucose-6-phosphatase

Derek P. Claxton[1*], Emily M. Overway[1], James K. Oeser[1], Richard M. O'Brien[1] and Hassane S. Mchaourab[1]

[1]From the Department of Molecular Physiology and Biophysics, Vanderbilt University, Nashville, TN 37232, USA

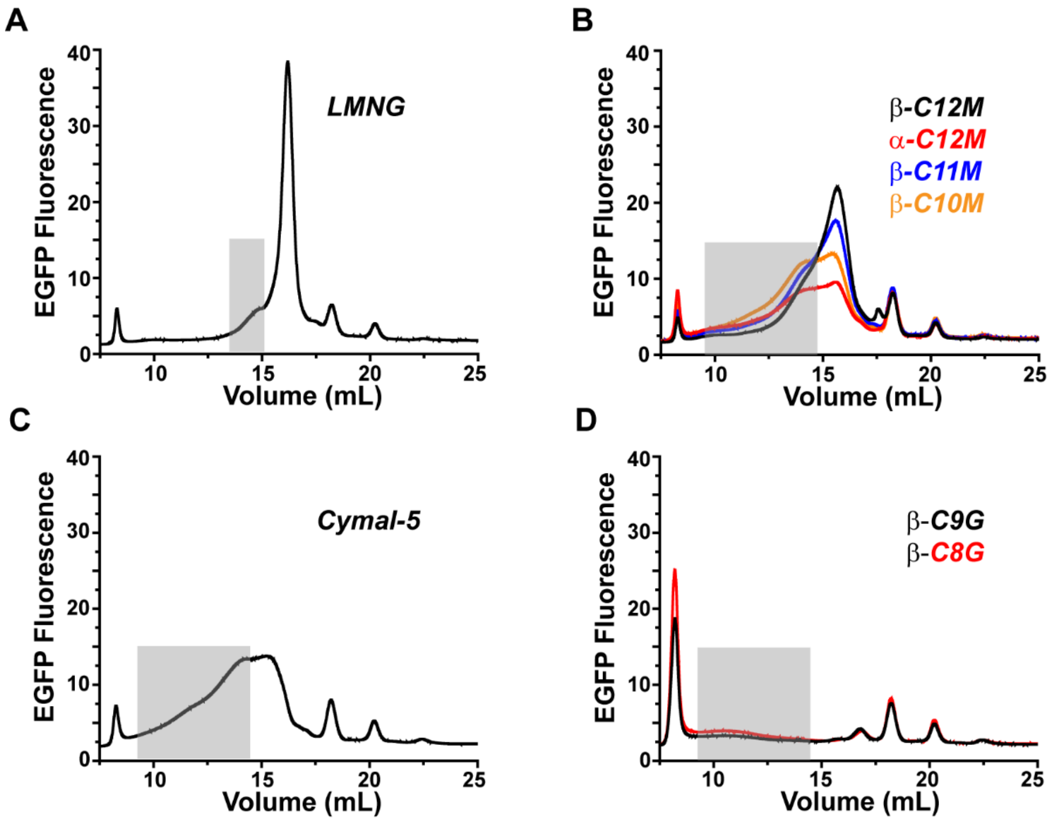

Figure S1. Preliminary detergent solubilization screen of mG6PC1-EGFP expressed in HEK293S cells. A representative panel of detergents with unique physiochemical properties were chosen for whole-cell solubilization of mammalian cells expressing the mouse fusion construct. The FSEC traces are grouped according to detergent class (**A**, neopentyl glycol; **B**, maltosides; **C**, cymal and **D**, glucosides). Detergent concentrations for extraction were 5 mM (neopentyl glycol), 40 mM (maltosides and Cymal-5), 60 mM (β-C9G) and 100 mM (β-C8G). Solubilization proceeded as described in the **Experimental Procedures**. The traces were assessed for relative homogeneity and the presence of broad, heterogeneous components indicative of protein aggregation (shaded region). LMNG (**A**) and β-C12M (**B**) displayed the most favorable traces according to this qualitative metric. However, all other detergents tested increased the pool of aggregated species relative to LMNG and β-C12M.

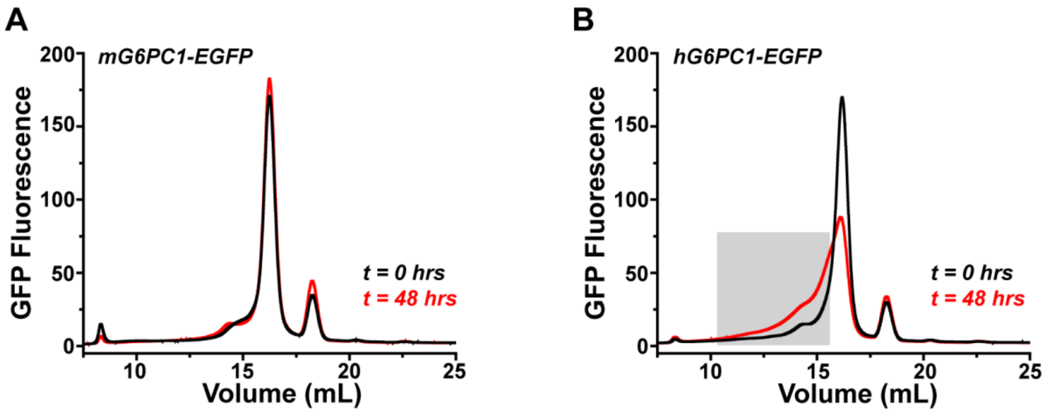

Figure S2. FSEC analysis of mouse and human G6PC1 solubilized in LMNG micelles. Whereas the elution profile of mG6PC1-EGFP remains relatively homogeneous over a 48 hr period (**A**), hG6PC1-EGFP displays characteristics of aggregation (shaded region, **B**) over the same time period. The data indicates that hG6PC1-EGFP is not as stable as mG6PC1-EGFP.

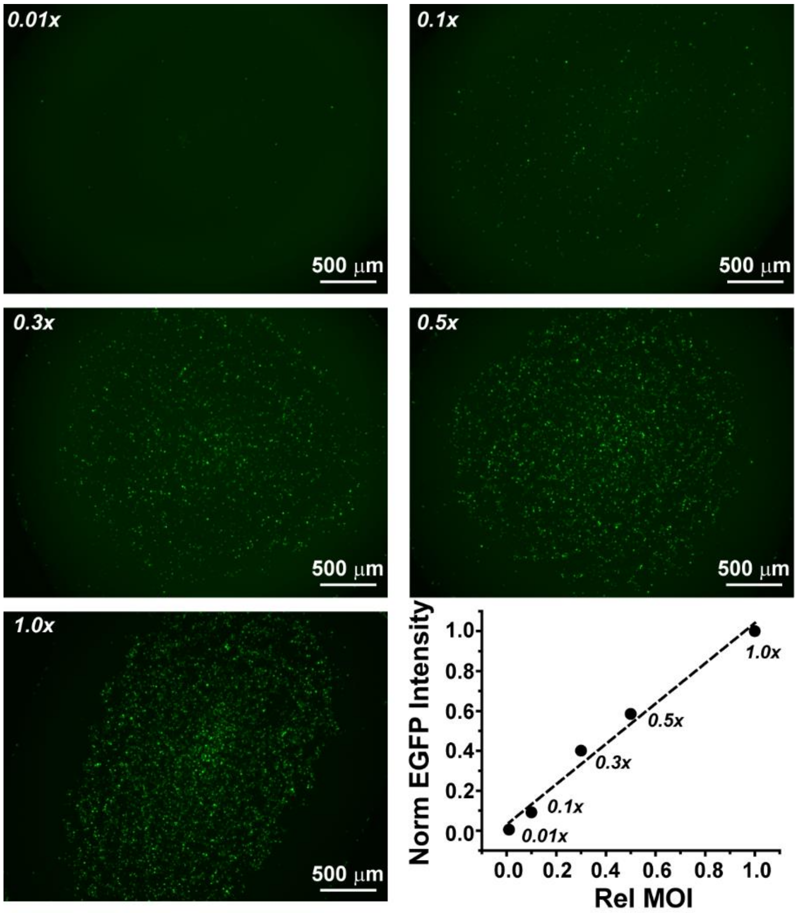

Figure S3. Sf9 cell epifluorescence following transduction of baculovirus carrying mG6PC1-EGFP. The images capture differences in fusion protein expression as a function of viral load (Rel MOI) after 24 hrs at 27 °C. The total fluorescence of each image was quantified in ImageJ software and plotted as a function of Rel MOI (bottom right panel). The dashed line is a linear fit of the data (slope = 1.01). The corresponding plot generated from FSEC analysis of LMNG-solubilized Sf9 cells is shown in Fig 2A.

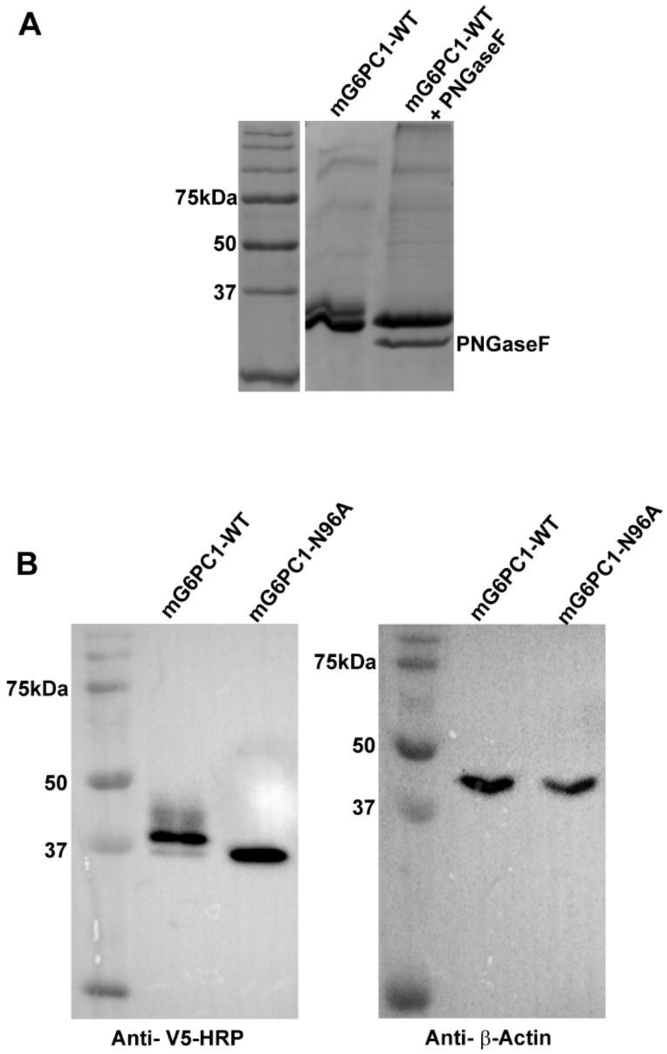

Figure S4. mG6PC1-WT is glycosylated at Asn96. (A) Treatment of purified mG6PC1-WT with PNGaseF under denaturing conditions induces collapse of the doublet to a single band. (**B**) Representative Western blots from transient transfection of pJPA5 plasmids encoding mG6PC1-WT or mG6PC1-N96A shows that glycosylation of the WT construct approached 90% in the INS-1 832/13 cell line. Although expression of WT and N96A is similar, glycosylation is lost in the N96A mutant.

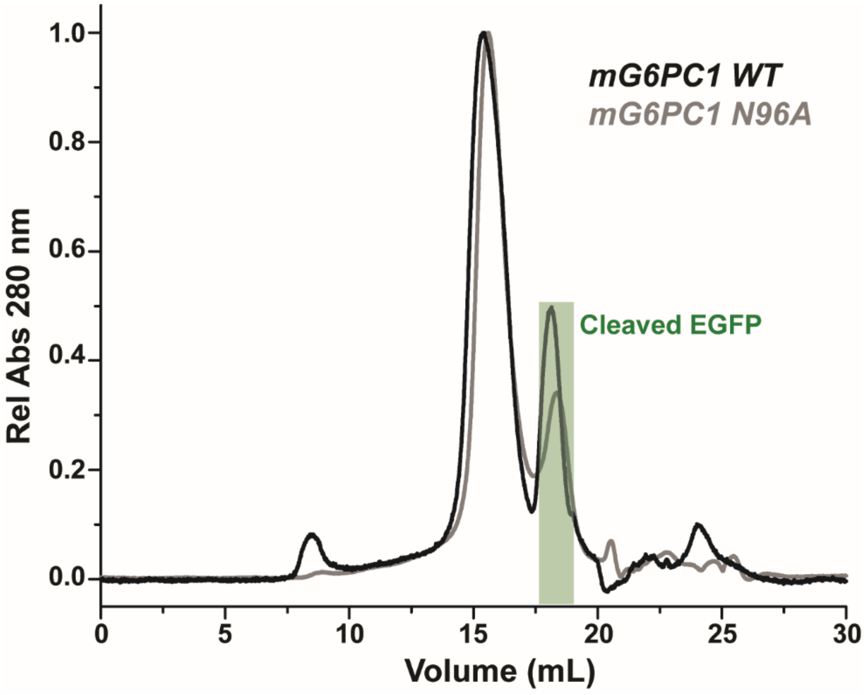

Figure S5. Preparative size exclusion chromatography of mG6PC1-WT and -N96A in LMNG micelles. Cleaved EGFP obtained from the thrombin protease reaction is removed efficiently by the SEC step to yield homogeneous preparations of mG6PC1-WT or the N96A variant. The corresponding analytical FSEC traces monitoring Trp fluorescence are shown in Fig 3B.

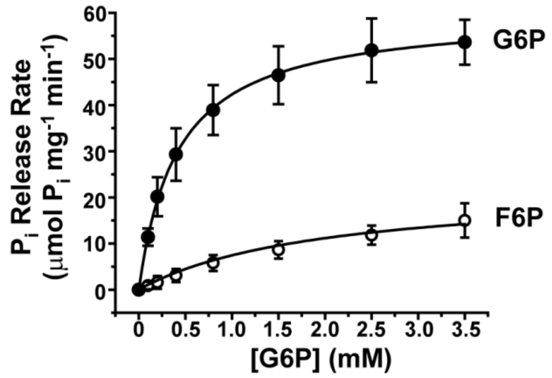

Substrate	K_M (mM)[a]	V_{max} (μmol P_i mg^{-1} min^{-1})[a]	k_{cat} (sec^{-1})	k_{cat}/K_M (M^{-1} sec^{-1})
G6P	0.431 ± 0.08	60.14 ± 5.39	41	9.6×10^4
F6P	1.833 ± 0.29	20.93 ± 4.03	14	7.9×10^3

[a] *n* = 3-4 replicates

Figure S6. Comparison of phosphohydrolase activity between G6P and F6P. The kinetics of P_i release were measured under standard conditions at 30 °C for 1 min in pH 6.5 buffer. Purified mG6PC1 is an order of magnitude more efficient in the hydrolysis of G6P relative to F6P. The data reported is the mean ± standard deviation.

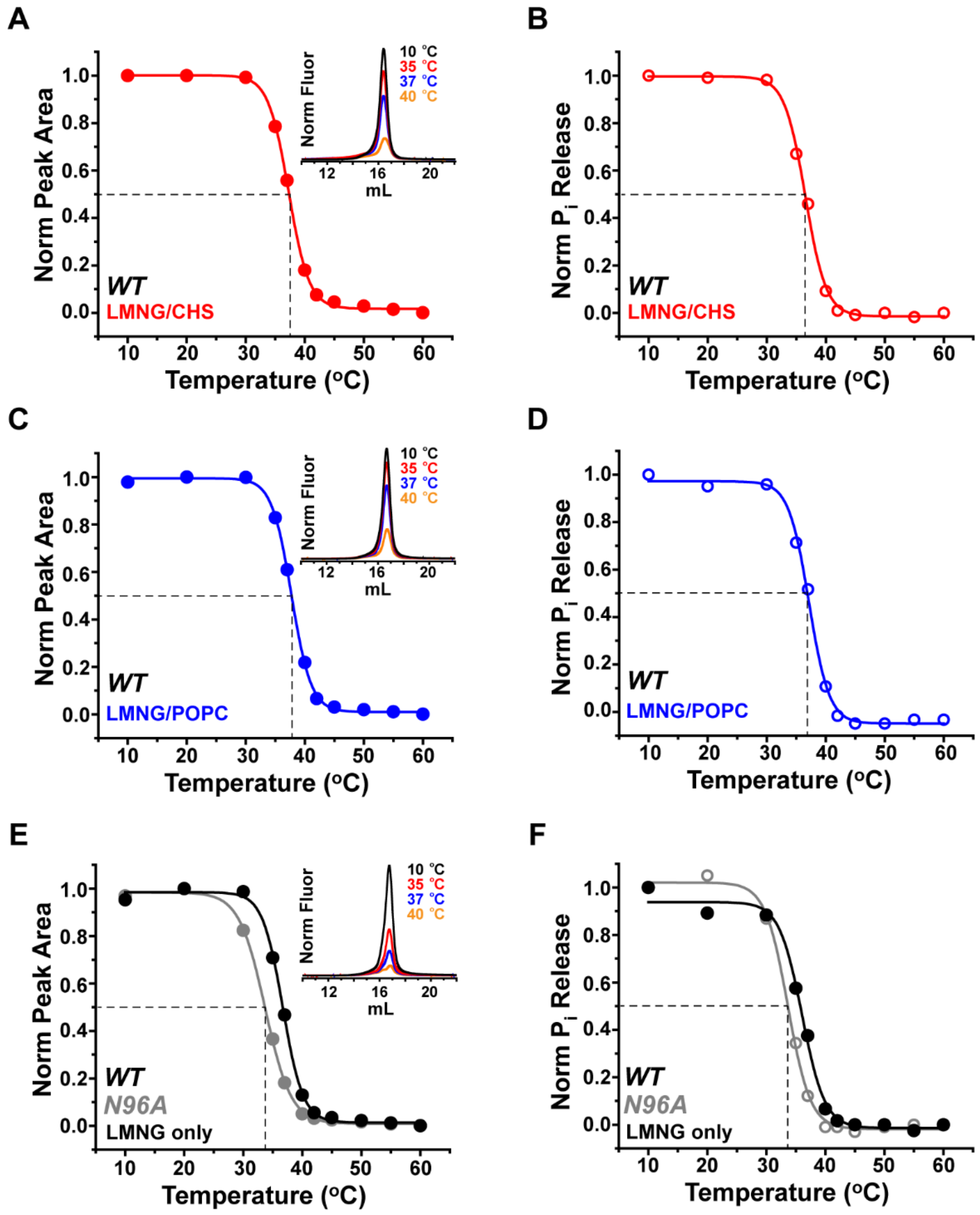

Figure S7. Thermostability analysis of mG6PC1-WT and the –N96A variant. Panels (**A**), (**C**) and (**E**) are melt curves derived from the FSEC analysis (inset), whereas the corresponding panels (**B**), (**D**) and (**F**) were obtained from activity assays. Panels (**A-B**) and (**C-D**) report the thermostabilizing effect of supplementing LMNG micelles with CHS and POPC, respectively, for mG6PC1-WT. Panels (**E-F**) illustrate the destabilization of the N96A mutant (LMNG micelles alone) relative to WT.

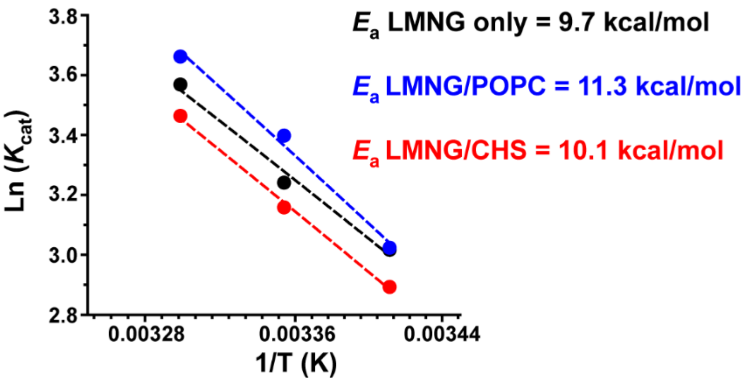

Figure S8. Determination of mG6PC1 activation energy (E_a) purified in three micellar conditions. The temperature dependence of k_{cat}, derived from velocity curves, was transformed into Arrhenius plots. Slopes of the linear regressions (dashed lines) report that E_a is similar in all conditions with an average E_a of 10.4 ± 0.8 kcal/mol.
